# Secreted heme peroxidase from *Dictyostelium discoideum*: Insights into catalysis, structure, and biological role

**DOI:** 10.1074/jbc.RA117.000463

**Published:** 2017-12-14

**Authors:** Andrea Nicolussi, Joe Dan Dunn, Georg Mlynek, Marzia Bellei, Marcel Zamocky, Gianantonio Battistuzzi, Kristina Djinović-Carugo, Paul G. Furtmüller, Thierry Soldati, Christian Obinger

**Affiliations:** From the ‡ Department of Chemistry, Division of Biochemistry, BOKU-University of Natural Resources and Life Sciences, 1190 Vienna, Austria,; the § Department of Biochemistry, Faculty of Science, University of Geneva, 1211 Genève, Switzerland,; the ¶ Department for Structural and Computational Biology, Max F. Perutz Laboratories, University of Vienna, 1030 Vienna, Austria,; the Departments of ‖ Life Sciences and; ‡‡ Chemistry and Geology, University of Modena and Reggio Emilia, via Campi 103, 41125 Modena, Italy,; the ** Institute of Molecular Biology, Slovak Academy of Sciences, 84551 Bratislava, Slovakia, and; the §§ Department of Biochemistry, Faculty of Chemistry and Chemical Technology, University of Ljubljana, 1000 Ljubljana, Slovenia

**Keywords:** Dictyostelium, post-translational modification (PTM), heme, peroxidase, heme peroxidase, oxidation-reduction (redox), X-ray crystallography, kinetics, antibacterial activity, halide oxidation, modified heme-modified heme, antibacterial activity, fruiting body

## Abstract

Oxidation of halides and thiocyanate by heme peroxidases to antimicrobial oxidants is an important cornerstone in the innate immune system of mammals. Interestingly, phylogenetic and physiological studies suggest that homologous peroxidases are already present in mycetozoan eukaryotes such as *Dictyostelium discoideum*. This social amoeba kills bacteria via phagocytosis for nutrient acquisition at its single-cell stage and for antibacterial defense at its multicellular stages. Here, we demonstrate that peroxidase A from *D. discoideum* (DdPoxA) is a stable, monomeric, glycosylated, and secreted heme peroxidase with homology to mammalian peroxidases. The first crystal structure (2.5 Å resolution) of a mycetozoan peroxidase of this superfamily shows the presence of a post-translationally-modified heme with one single covalent ester bond between the 1-methyl heme substituent and Glu-236. The metalloprotein follows the halogenation cycle, whereby compound I oxidizes iodide and thiocyanate at high rates (>10^8^ m^−1^ s^−1^) and bromide at very low rates. It is demonstrated that DdPoxA is up-regulated and likely secreted at late multicellular development stages of *D. discoideum* when migrating slugs differentiate into fruiting bodies that contain persistent spores on top of a cellular stalk. Expression of DdPoxA is shown to restrict bacterial contamination of fruiting bodies. Structure and function of DdPoxA are compared with evolutionary-related mammalian peroxidases in the context of non-specific immune defense.

## Introduction

For more than 70 years, *Dictyostelium discoideum* has been used as a model organism for various fundamental biological processes such as phagocytosis, autophagy, cell aggregation, or cell communication (1–3). As a single cellular amoeba, *D. discoideum* lives as a professional phagocyte, feeding on bacteria. In its natural habitat, *D. discoideum* is able to engulf, kill, and digest microorganisms at a rate of at least one/min (2). Upon starvation, it undergoes a program of multicellular development, leading to differentiation into fruiting bodies containing persistent spores (4, 5). Both the single-celled amoebae and the multicellular aggregates have developed antibacterial defense mechanisms that exhibit many parallels to mammalian innate immune responses, including phagocytosis by many types of white blood cells. *D. discoideum* as a model system has shed light on the conserved molecular mechanisms of phagocytosis and the evolution of innate immune responses (2).

Sequencing and annotation of the genome of *D. discoideum* (Dd) revealed the presence of three peroxidases, namely one representative of the peroxiredoxin family (*i.e.* peroxiredoxin-4, prdx4) and two heme peroxidases, namely DdPoxA and DdPoxB from two different superfamilies (6, 7). DdPoxB belongs to class B of the family of so-called dye-decolorizing peroxidases (8), whereas DdPoxA is a member of the peroxidase–cyclooxygenase superfamily (7, 9). Most interestingly, DdPoxA shares homology with mammalian peroxidases, which are important players in the mammalian innate immune response (9).

The peroxidase–cyclooxygenase superfamily has been shown to be composed of seven families (7, 9). Family 1 is composed of chordata peroxidases, including thyroid peroxidase, lactoperoxidase (LPO),^4^ eosinophil peroxidase (EPO), and myeloperoxidase (MPO). LPO, EPO, and MPO play an antimicrobial role by catalyzing the production of reactive oxidants, *e.g.* hypohalous acids or hypothiocyanate (10, 11). LPO is secreted from mammary, salivary, and other mucosal glands; EPO is released by eosinophils, and MPO is secreted into the phagolysosome of phagocytosing neutrophils upon degranulation to kill engulfed pathogens such as bacteria (12–14).

Phylogenetic analysis demonstrated that DdPoxA belongs to family 6 of this heme peroxidase superfamily, shares a striking sequence similarity to the mammalian heme peroxidases, and has a signal peptide for secretion (7). The substantial similarity of the molecular mechanism(s) of phagocytosis and bacterial killing for food acquisition in *D. discoideum* and of the antimicrobial activity of neutrophils, monocytes, or macrophages (15, 16) prompted us to investigate the biochemistry and physiology of DdPoxA.

Here, we present the biochemical characterization and the first crystal structure of DdPoxA and demonstrate that the overall structures and heme cavity architecture of mammalian peroxidases were already established very early in evolution. Similar to family 1 peroxidases, the heme of DdPoxA is post-translationally modified by an autocatalytic process. In contrast to the mammalian enzymes (17–19), only one heme to protein ester bond is found, which is formed between Glu-236 and the 1-methyl substituent of the porphyrin ring. The metalloenzyme is shown to follow the halogenation cycle and to catalyze the efficient two-electron oxidation of iodide and thiocyanate. Nevertheless, DdPoxA is a poor oxidizer of bromide and cannot mediate the oxidation of chloride. The presented *in vivo* investigations include a detailed expression pattern of *DdpoxA* throughout the development cycle of *D. discoideum* at the protein level together with comparative cell development studies on wildtype *D. discoideum* and the *DdpoxA* knockout mutant (Δ*DdpoxA*). It is demonstrated that the heme enzyme supports maintenance of sterility of the slug and the subsequently produced fruiting bodies, which suggests a role in antibacterial defense of the multicellular aggregate.

## Results

### Recombinant DdPoxA is a stable, monomeric, and glycosylated heme peroxidase

**Figure 1.**
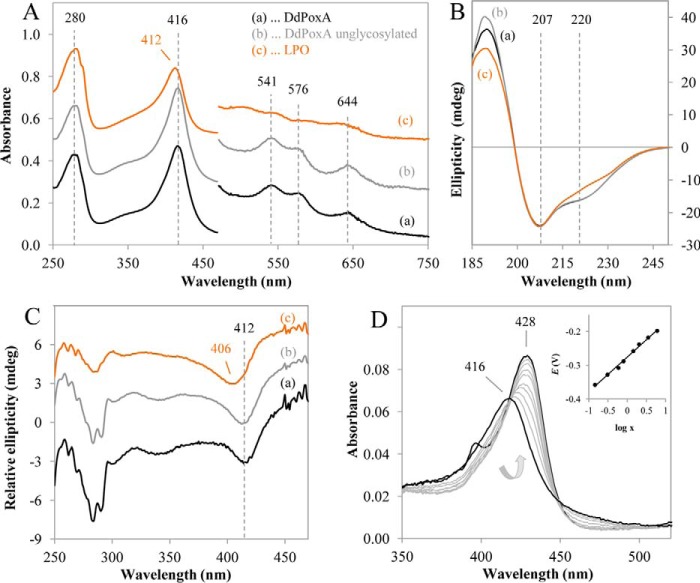
**UV-visible ECD spectra and reduction potential of DdPoxA.** *A,* UV-visible spectra of purified DdPoxA and LPO were recorded using 5 μm protein in 50 mm phosphate buffer at pH 7.0. The wavelength range from 480 to 750 nm is expanded by a factor of 5 for better visibility. *B,* electronic circular dichroism spectra of DdPoxA in the far-UV region from 185 to 250 nm at 25 °C. Concentration of DdPoxA was 0.5 mg/ml in 10 mm phosphate buffer (pH 7.0). The path length was 1 mm, with a bandwidth of 3 nm, using a scan time of 10 s per point. *C,* electronic circular dichroism spectra of 10 μm DdPoxA and LPO in 50 mm phosphate buffer (pH 7.0) in the visible region from 250 to 470 nm at 25 °C. The path length was 10 mm; the spectral bandwidth was 1 nm, and the scan time was 10 s per point. *D,* electronic absorption spectra of DdPoxA at different applied potentials, whereby the *black lines* represent fully oxidized form (*A*_λox_ maximum at 416 nm) and fully reduced form (*A*_λred_ maximum at 428 nm). *Arrow* indicates the transition from oxidized to reduced form. Titration was performed with 10 μm protein in 100 mm potassium phosphate buffer (pH 7.0) containing 100 mm NaCl at 25 °C. *Inset,* corresponding Nernst plot, whereby *X* represents (*A*_428 nm_ maximum − *A*_428 nm_)/(*A*_410 nm_ maximum − *A*_410 nm_).

Recombinant DdPoxA was successfully produced with an N-terminal His_6_ tag in the *Pichia pastoris* BG11 (ΔAOX1) strain. The secreted protein was purified by a two-step ammonium sulfate precipitation and, after resuspension, by affinity chromatography. The yield was found to be ∼50 mg/liter of *P. pastoris* supernatant and the purity number (RZ, *A*_Soret_/*A*_280 nm_) 1.1–1.2 indicating 90–95% heme occupancy. Fig. 1*A* depicts the UV-visible spectrum of ferric DdPoxA with the Soret maximum at 416 nm, Q-bands at 541 and 576 nm, and the charge transfer band at 644 nm (black spectrum).

The secreted glycosylated peroxidase has three *N*-glycosylation sites at positions Asn-62, Asn-131, and Asn-338 as predicted by the on-line tool N-Glycosite (20). Analysis of the protein by SDS-PAGE and Western blotting showed hyperglycosylation, which is typical for recombinant protein expressed in *P. pastoris* (data not shown). Thus, deglycosylation of recombinant DdPoxA by Endo H_f_ glucosidase was performed, followed by SEC purification. Deglycosylation had no impact on the UV-visible and ECD spectral features in the far-UV, near-UV, and visible range, clearly suggesting that the overall and heme cavity architecture were not affected (compare *black* and *gray* spectra in Fig. 1, *A–C*). Between pH 5.0 and pH 9.0, the spectral features of DdPoxA remained unchanged (data not shown).

In the homologous lactoperoxidase (LPO) (*orange spectrum* in Fig. 1, *A–C*) the Soret band is blue-shifted as is evident by the respective UV-visible (412 nm) and ECD (minimum at 406 nm) spectra, which clearly suggest differences in the heme cavity architecture compared with DdPoxA. It is well known that LPO has two heme to protein ester bonds derived from a hydrogen peroxide-mediated post-translational modification (18, 22). The involved acidic amino acids (Asp-225 and Glu-375, goat LPO numbering) are fully conserved in family 1 peroxidases (Fig. S1). Interestingly, the sequence alignment shown in Fig. S1 suggests that DdPoxA has only one covalent link between the 1-methyl substituent of the prosthetic group and Glu-236, because the DdPoxA residue corresponding to Asp-225 of LPO is an isoleucine.

Differences in the active-site architecture were also reflected by spectroelectrochemical investigations of DdPoxA at pH 7.0. Upon reduction of ferric to ferrous DdPoxA, its Soret peak shifts from 416 to 428 nm. Fig. 1*D* shows a representative family of spectra at different applied potentials in the OTTLE cell. The calculated reduction potential, *E*′^0^, for the Fe(III)/Fe(II) couple, determined from the corresponding Nernst plot (*inset* to Fig. 1*D*), was calculated to be −0.276 ± 0.010 V, which is significantly more negative compared with LPO (−0.176 V) (23).

Importantly, incubation of recombinant DdPoxA with low micromolar hydrogen peroxide altered neither its UV-visible and ECD spectral features nor the standard reduction potential (data not shown). This suggests that the autocatalytic post-translational modification of the heme group (24) was already established during recombinant protein production in the methylotrophic yeast. By contrast, for other members of this heme peroxidase superfamily addition of excess H_2_O_2_ was necessary to complete the post-translational modification, which was reflected by changes in spectral and redox properties (25–28).

**Figure 2.**
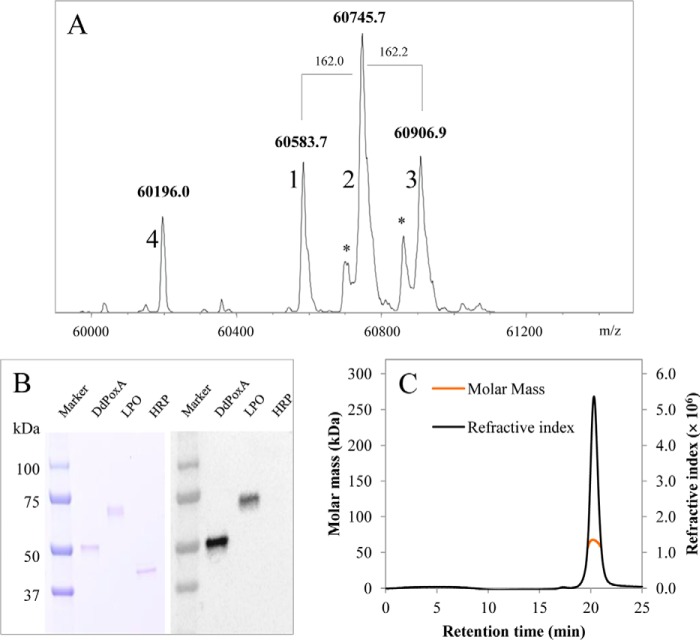
**Evidence of covalently bound heme and analysis of oligomeric state.** *A,* for intact mass spectrometry, 2.5 μg of DdPoxA in 10 mm phosphate buffer (pH 7.0) was directly injected to the LC-MS system and analyzed by a QTOF instrument equipped with the standard ESI source in the positive ion mode. *Asterisks* represent non-specific adducts caused by TFA. *B,* SDS-PAGE (*left panel*) and detection of covalently bound heme by ECL (*right panel*) of 0.3 μg of deglycosylated DdPoxA (*lane 1*), lactoperoxidase (*lane 2*), and horseradish peroxidase (*lane 3*). *C,* presentation of MALS analysis showing refractive index (*black line*) detection. The *orange line* shows the distribution of molar masses in the analyzed protein solution.

Next, we probed the presence of the proposed covalent heme to protein ester bond in deglycosylated DdPoxA by mass spectrometry (Fig. 2*A*). The resulting chromatogram shows four major peaks, whereby peak 1 (60,583.7 Da) was assigned to DdPoxA with three *N*-acetyl-d-glucosamines (GlcNAcs) and a covalently bound heme. Peaks 2 and 3 in the chromatogram (60,745.7 and 60,906.9 Da) represent DdPoxA carrying one and two additional hexoses (162 Da each). Peptide mapping revealed that these hexoses derive from single *O-*glycosylations at positions Thr-133 and Thr-142, which were not cleaved during deglycosylation (data not shown). The minor peak 4 (60,196 Da) was assigned to a degradation product lacking four histidines from the N-terminal His_6_ tag. Most importantly, no peak could be assigned to protein without covalently bound heme.

The presence of a covalently bound heme in DdPoxA was further confirmed by SDS-PAGE, and visualization of the blotted proteins was by enhanced chemiluminescence. Note that unbound heme is lost during SDS-PAGE and Western blotting. Fig. 2*B* compares LPO, DdPoxA, and horseradish peroxidase (HRP). The latter carries non-covalently bound heme *b* at the active site. It is clearly demonstrated that both LPO and DdPoxA but not HRP exhibited chemiluminescence at the respective protein bands, demonstrating covalent heme attachment.

Furthermore, we probed the oligomeric state of deglycosylated DdPoxA by SEC-MALS. Fig. 2*C* depicts a sharp peak at a retention time at 20.3 min in the corresponding chromatogram. By using MALS, an average molar mass of 63.4 kDa was calculated, which is similar to the theoretical mass (60.7 kDa) and suggests the presence of a monomer.

**Figure 3.**
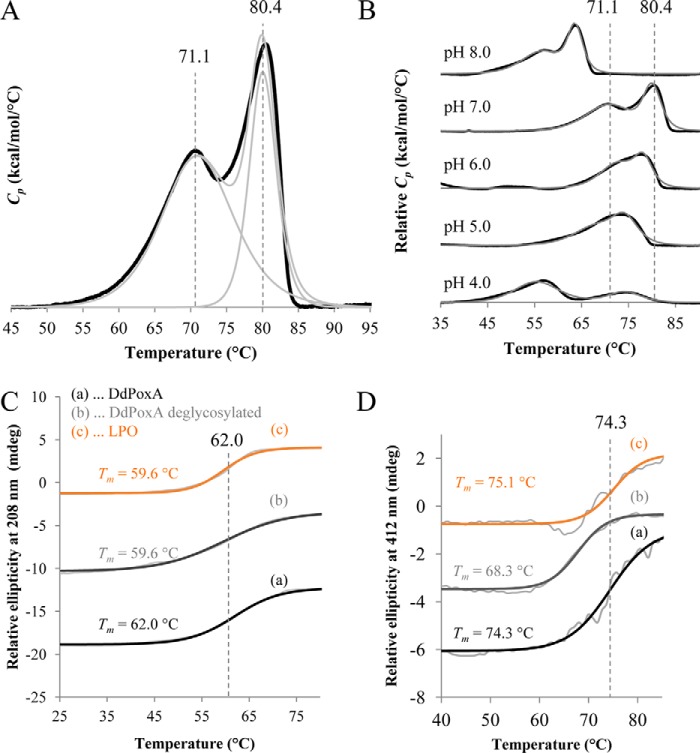
**Investigation of thermal and conformational stability of DdPoxA.** *A* and *B*, normalized DSC thermograms of DdPoxA at pH 7.0 (*A*) and pH 4.0–8.0 (*B*) after baseline correction. Measurements were performed with 5 μm protein in 100 mm phosphate buffer, using a heat rate of 60 °C/h. The obtained thermograms are shown as *bold black lines,* and the corresponding fitted non-two-state transition peaks are depicted as *thin gray lines. Vertical lines* have been inserted for presentation of respective melting temperatures at pH 7.0. *C,* temperature-mediated unfolding of DdPoxA was monitored by electronic circular dichroism at 208 nm, following the melting of α-helical structures in 100 mm phosphate buffer (pH 7.0). *D,* temperature-mediated unfolding of DdPoxA monitored by electronic circular dichroism at 412 nm in 100 mm phosphate buffer (pH 7.0), showing the unfolding of the heme cavity.

Finally, we investigated the thermal stability of DdPoxA using differential scanning calorimetry and temperature-dependent ECD experiments (Fig. 3). In the DSC experiment, ferric glycosylated DdPoxA showed two independent unfolding events with *T_m_* values of 71.1 and 80.4 °C, respectively, at pH 7.0 (Fig. 3*A*). The thermal stability rapidly decreases at alkaline pH, whereas stability is retained in the acidic region until pH 5.0 (Fig. 3*B*).

Complementary temperature-dependent ECD measurements of glycosylated DdPoxA at pH 7.0 showed a mid-point transition of 62 °C in the far-UV at 208 nm reflecting unfolding of the secondary structure (Fig. 3*C*). The overall secondary structures of deglycosylated DdPoxA and LPO were slightly less stable (*T_m_* = 59.6 °C). In the visible region at 408 nm, glycosylated DdPoxA showed a *T_m_* value of 74.3 °C suggesting that the second endotherm in the DSC thermogram reflects unfolding of the heme cavity (Fig. 3*D*). The corresponding *T_m_* values of deglycosylated DdPoxA and LPO were found to be at 68.3 and 75.1 °C, respectively.

### DdPoxA displays high structural similarity to mammalian peroxidases but unusual post-translational heme modification

**Figure 4.**
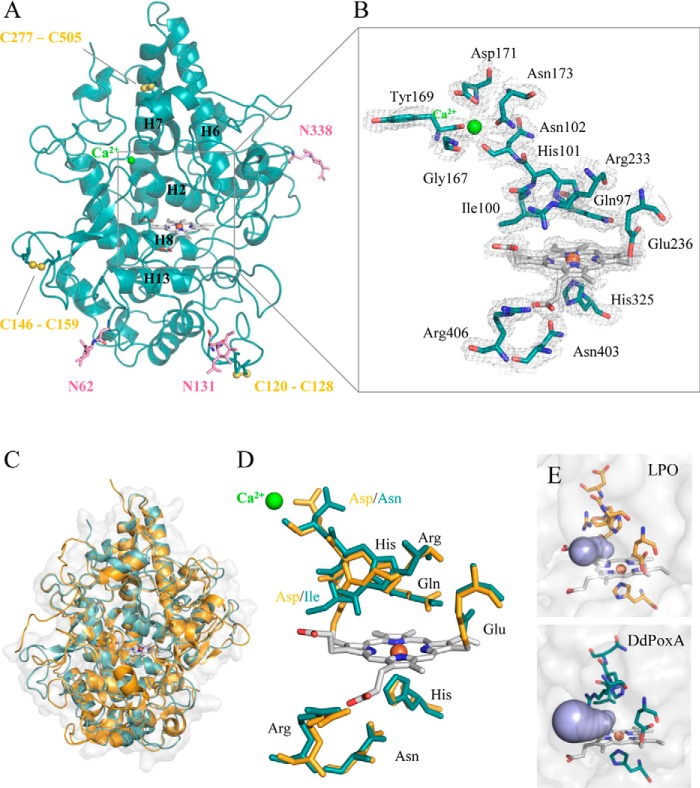
**Structural investigation of DdPoxA.** *A,* crystal structure of recombinant DdPoxA (in *teal*, PDB accession code 6ERC). Chain A of the asymmetric unit was used for preparation of figures. The three disulfide bridges are depicted in *yellow*. The three *N*-glycosylation sites with one attached GlcNAc are shown in *pink*; the distal Ca^2+^ ion is highlighted in *green*. Important core helices are indicated, including their corresponding numbering. *B,* close-up of the active site and the Ca^2+^-binding site of DdPoxA, including a 2.5 Å resolution FEM map contoured at 1 σ (*gray*). *C,* overlay of the secondary structures of DdPoxA (*teal*) and goat LPO (*yellow,* PDB accession code 2R5L). *D,* active-site overlay of DdPoxA (*teal*) and goat LPO (*yellow*). *E,* accessibility of substrate channels of LPO (*yellow*) and DdPoxA (*teal*) with the highest calculated probabilities. Substrate channels are depicted in *violet*. Distances and bottlenecks were calculated with CAVER 3.0.

Crystal structures of deglycosylated DdPoxA (space group P 3_1_ 2 1) (Table 1) comprises two DdPoxA molecules per asymmetric unit (r.m.s.d. value between the two chains: 0.54 Å calculated over 508 C_α_ atoms, PDB code 6ERC). The structure was solved to 2.5 Å resolution by molecular replacement using the balbes web server (29). Fig. 4*A* shows the overall largely α-helical structural organization of DdPoxA. Only three small antiparallel β-strands (residues Phe-43–Pro-46, Ala-141–Glu-143, and Tyr-160–Asn-164) are present. The protein is composed of 21 α-helices of varying length. The central core of the molecule consists of five long α-helices (H2, H6, H7, H8, and H13) with the covalently attached heme group. A very similar architecture is found in LPO (core helices are H2, H5, H6, H8, and H12) (r.m.s.d. value between the chain A of DdPoxA and goat LPO (PDB code 2R5L) is 1.61 Å calculated over 444 C_α_ atoms, *Z*-score is 13.4) (22). The conserved distal residues Gln-97 and His-101 are found in helix 2, whereas the proximal His-325 belongs to helix 8 and the proximal Asn-403 and Arg-406 are part of helix 13. As in LPO, the core helices form triangles (H6, H7, and H8 in DdPoxA and H5, H6, and H8 in LPO), whereby the heme group is located between helix 2 and 8 (22).

The heme environment of DdPoxA shows a striking sequence similarity to LPO (Fig. S1). The important catalytic residues on the distal heme site (His-101, Gln-97, and Arg-233) are fully conserved, including Glu-236 that forms an ester bond with the 1-methyl substituent of the heme porphyrin ring (Fig. 4*B* and Fig. S2). However, DdPoxA lacks the covalent ester bond that is formed between the heme and the distal aspartate in LPO, because the corresponding residue in DdPoxA is an isoleucine. The position of the distal Ca^2+^-binding site is conserved; however, different residues are involved in binding of the ion. In LPO, the Ca^2+^-binding site consists of the residues Asp, Asp, Ser, Tyr, and Phe, and in DdPoxA, the ion is coordinated by Asn-102, Gly-167, Tyr-169, Asp-171, and Asn-173. The proximal triad His-325, Asn-403, and Arg-406 is fully conserved between the two enzymes (Fig. 4, *C* and *D*).

The polypeptide chain of DdPoxA contains eight cysteines, six of which are involved in disulfide bridges (Cys-120–Cys-128, Cys-146–Cys-159, and Cys-277–Cys-505) (Fig. 4*A*). In comparison, LPO has 15 cysteine residues, forming seven intramolecular disulfide bridges. None of the disulfide bridges are conserved between DdPoxA and LPO. DdPoxA contains three *N*-glycosylation sites (Asn-62, Asn-131, and Asn-338), which are all occupied by complex sugar moieties in the recombinant protein. However, in the crystal structure, only one GlcNAc is left on each *N*-glycosylation site due to the deglycosylation procedure (see also Fig. 2*A*). In comparison, LPO shows four *N*-glycosylation sites, and none of them is conserved between the two proteins.

Approximate bottlenecks of the substrate channel were determined by tunnel calculations using CAVER 3.0. The most important channels of both, DdPoxA and goat LPO, are given by throughput values of 0.75 and 0.79, respectively (throughput values range from 0 to 1; the higher the value, the greater the probability that the tunnel is functionally relevant). The bottleneck radii 1.19 Å (DdPoxA) and 1.22 Å (LPO) are very similar, but their length differs significantly with 8.61 Å (DdPoxA) compared with 4.85 Å (LPO) (Fig. 4*E*).

### DdPoxA compound I is an efficient oxidant of iodide and thiocyanate

**Figure 5.**
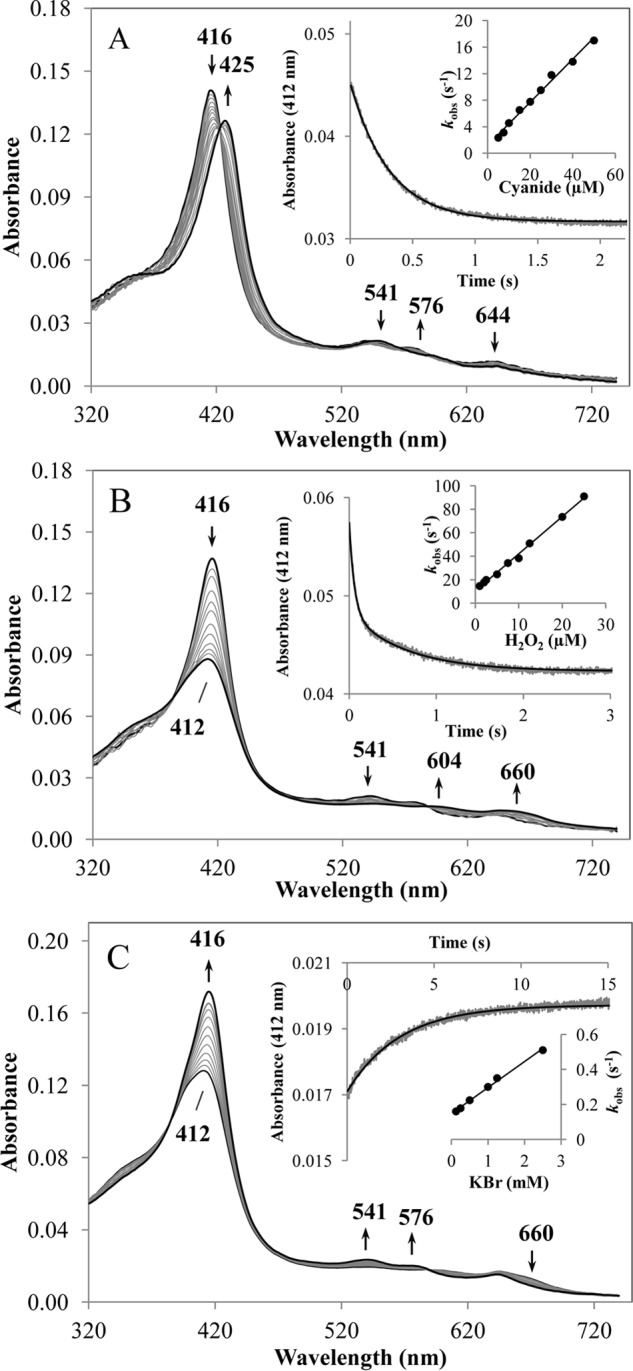
**Reaction of ferric DdPoxA with cyanide and H_2_O_2_ and reaction of compound I with bromide.** *A,* spectral transition upon binding of 500 μm cyanide to 2 μm DdPoxA, measured in conventional stopped-flow mode. The first spectrum shows the ferric protein in its high-spin state (absorbance maximum at 416 nm); the second spectrum was recorded after 5 ms of mixing, and the following spectra show the formation of the DdPoxA–cyanide low-spin complex (absorbance maximum at 425 nm). *Arrows* indicate directions of spectral changes. All measurements were performed in 50 mm phosphate buffer (pH 7.0) at 25 °C. *Main inset,* typical time trace at 412 nm upon reaction of 1 μm protein with 7.5 μm cyanide (single exponentially fitted). *Inset within inset,* linear dependence of *k*_obs_ from cyanide concentrations. The apparent association rate constant *k*_on_ was obtained from the slope and the apparent dissociation rate constant *k*_off_ was calculated from the intercept. The dissociation constant *K_D_* is determined by the ratio of *k*_off_/*k*_on_. *B,* spectral changes during reaction of 2 μm DdPoxA with 5 μm H_2_O_2_ measured in the conventional stopped-flow mode. The first spectrum shows the ferric protein; the second spectrum was recorded 1 ms after mixing, and the subsequent spectra display the transition to compound I. *Main inset,* typical time trace at a wavelength of 412 nm upon reaction of 0.5 μm DdPoxA with 2 μm H_2_O_2_, fitted as a double exponential function. *Inset within inset,* linear dependence of *k*_obs_ from H_2_O_2_ concentrations. The apparent bimolecular rate constant *k*_app_ was obtained from the slopes of the regression lines. *C,* spectral transition upon adding 5 mm bromide to 2 μm DdPoxA compound I in the sequential stopped-flow mode. Compound I was preformed using 5 μm hydrogen peroxide. After a delay time of 400 ms, the first spectrum was recorded 4.9 s after mixing. Subsequent spectra show the reduction of compound I. *Main inset,* typical time trace at 412 nm of 0.5 μm compound I with 5 mm bromide (single exponential fit). *Inset within inset,* linear dependence of the pseudo-first-order constant *k*_obs_ from bromide concentrations.

Finally, we probed the accessibility and binding kinetics of cyanide to the heme cavity of ferric DdPoxA. Upon cyanide binding, the high-spin (*S* = 5/2) ferric protein is converted into the low-spin ferric protein (*S* = 1/2), thereby shifting the Soret maximum from 416 to 425 nm with a clear isosbestic point at 420 nm. In the visible range, a typical low-spin peak at 541 nm arose, whereas the peaks at 576 and 644 nm disappeared (Fig. 5*A*). Plotting the single exponentially fitted pseudo-first order rate constants (*k*_obs_) *versus* cyanide concentration yielded an apparent second-order dissociation rate constant (*k*_on_) of 3.3 × 10^5^ m^−1^ s^−1^ (*K_D_ = k*_off_/*k*_on_ = 3.7 μm), which was ∼5-fold lower than for MPO (*k*_on_ = 1.6 × 10^6^ m^−1^ s^−1^, *K_D_* = 1.9 μm) and LPO (*k*_on_ = 1.3 × 10^6^ m^−1^ s^−1^, *K_D_* = 23 μm) (17, 30–32). At pH 5.0, cyanide binding to ferric DdPoxA decreased (*k*_on_ = 8.9 × 10^4^ m^−1^ s^−1^ and *K_D_ = k*_off_/*k*_on_ = 10.1 μm).

Next, we tested the reaction of ferric DdPoxA with H_2_O_2_. Formation of compound I was characterized by a decrease of the Soret maximum and a shift to 412 nm and the formation of typical peaks in the visible range at 604 and 660 nm, whereas the peak at 541 nm vanished (Fig. 5*B*). Full hypochromicity of the Soret peak (46%) was obtained using a 2.5-fold stoichiometric excess of H_2_O_2_. The reaction showed a biphasic behavior with the fast reaction being responsible for ∼85% of absorbance change at 412 nm. The apparent bimolecular rate constant was calculated to be 3.2 × 10^6^ m^−1^ s^−1^, which is ∼5 times slower compared with MPO and LPO (*k*_app_ = 1.1 -1.4 × 10^7^ m^−1^ s^−1^) (29). Similar to cyanide binding, the rate of compound I formation decreased at pH 5.0 (*k*_app_ = 5.9 × 10^5^ m^−1^ s^−1^).

Furthermore, we probed the reaction of the preformed DdPoxA compound I with the two-electron donors chloride, bromide, iodide, and the pseudohalide thiocyanate. Chloride was unable to reduce compound I, although bromide is a very poor electron donor with *k*_app_ being 15 m^−1^ s^−1^ (pH 7.0) and 13 m^−1^ s^−1^ (pH 5.0) (Fig. 5*C*). However, oxidation of iodide and thiocyanate by DdPoxA compound I is extremely efficient (*k*_app_ >10^8^ m^−1^ s^−1^ at pH 7.0). Similar values have been reported for LPO, whereas MPO shows a 10-fold lower oxidation capacity for iodide and thiocyanate (*k*_app_ ∼10^7^ m^−1^ s^−1^) (30). At pH 5.0, oxidation of thiocyanate is also extremely fast (*k*_app_ >10^8^ m^−1^ s^−1^), whereas iodide oxidation at pH 5.0 is drastically impaired (7.4 × 10^3^ m^−1^ s^−1^).

### DdPoxA is expressed at multicellular stages and supports fruiting body sterility

**Figure 6.**
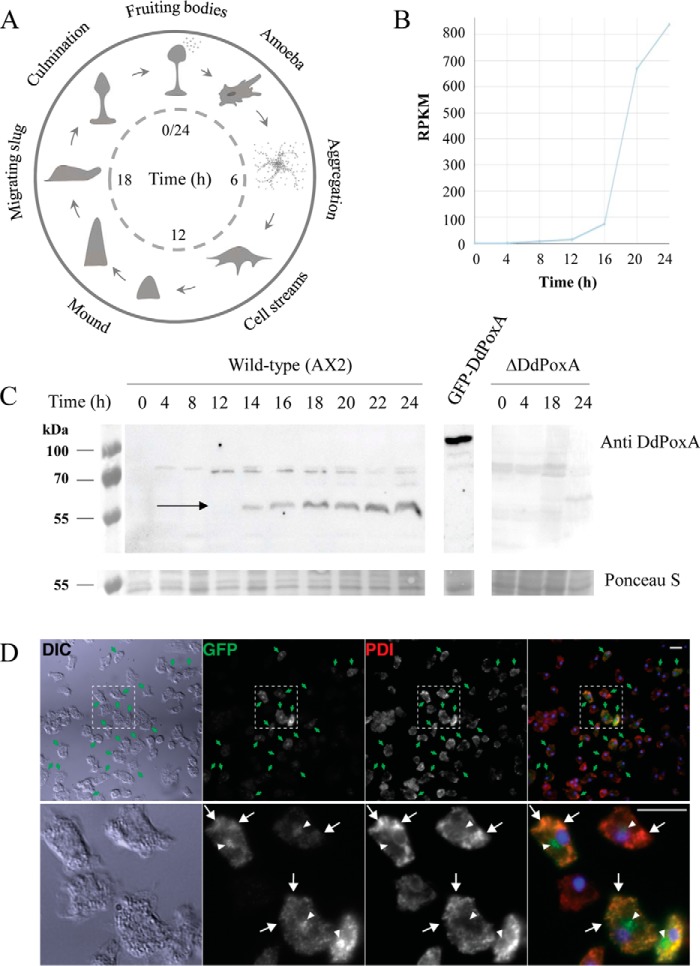
**Expression pattern of DdPoxA in *D. discoideum*.** *A,* schematic time course of *D. discoideum* development upon starvation in a time range of 24 h. *B,* open source RNAseq detection of DdPoxA throughout the development cycle of *D. discoideum* AX4 wildtype, whereby *RPKM* represents reads per million mapped reads. Data extracted using dictyExpress. *C,* Ponceau S staining and Western blotting of *D. discoideum* wildtype (AX2) and Δ*DdpoxA* cell lysates at different time points of development (0–24 h after start of starvation). Ponceau S staining and Western blotting detected by anti-actin indicate loading of equal protein concentrations. Anti-DdPoxA staining shows expression profile of DdPoxA upon cell development (*black arrow*). *D,* fluorescence microscopy analysis of DdPoxA localization. A heterogeneous population of *D. discoideum* expressing a GFP-tagged version of DdPoxA was fixed and stained with antibodies against GFP (*green*) and PDI (*red*), a marker for the endoplasmic reticulum. Nuclei were stained with DAPI (*blue*). *Scale bars,* 10 μm. In the *upper panel*, *green arrows* mark cells expressing PoxA-GFP, and the *box* delineates the area magnified in the *lower panel*, in which *white arrows* indicate co-localization in the ER, and *white arrowheads* indicate a Golgi-like staining pattern.

One remarkable characteristic of *D. discoideum* is its ability to shift from single cells to a multicellular stage in response to starvation. Starting from cell aggregation toward a cAMP signal, a multicellular aggregate is formed containing up to 100,000 cells. Subsequently, a migrating slug is formed that later culminates into a fruiting body, which comprises a spore-containing structure, the sorus, on top of a cellular stalk (5). Because the proteome and the morphology of the cells significantly change upon development, it is crucial to know the expression pattern of the investigated protein throughout the developmental cycle. Fig. 6*A* shows a simplified scheme of the developmental cycle of *D. discoideum.* Formerly published open source RNAseq data (33, 34) on the *D. discoideum* laboratory strain AX4 indicated that expression of DdPoxA was up-regulated at late developmental stages, starting approximately from 12 h after initiation of starvation (Fig. 6*B*). To confirm these findings, we let *D. discoideum* wildtype AX2 and Δ*DdpoxA* cells undergo the developmental cycle, and we detected the expression of DdPoxA by Western blotting with a polyclonal antibody against the recombinantly produced DdPoxA (Fig. 6*C*). The bands at ∼60 kDa confirmed the RNAseq data and clearly showed that the expression of DdPoxA was up-regulated at late developmental stages, ∼14 h after the initiation of starvation (Fig. 6*C, left panel*). A GFP-tagged version of DdPoxA serves as positive control (Fig. 6*C, middle panel*) and the Δ*DdpoxA* knockout mutant as negative control (*right panel*).

As outlined above, analysis of the polypeptide sequence suggested the existence of a signal peptide for secretion into the extracellular space. Fluorescence microscopy of a *D. discoideum* population expressing a GFP-tagged version of DdPoxA indicated that the tagged peroxidase co-localizes with the ER marker PDI (*arrows,* Fig. 6*D*). The additional juxtanuclear localization (*arrowheads,* Fig. 6*D*) is typical of the Golgi apparatus and/or the recycling endosomes (35, 36), suggesting that DdPoxA is most probably present in the secretory pathway.

**Figure 7.**
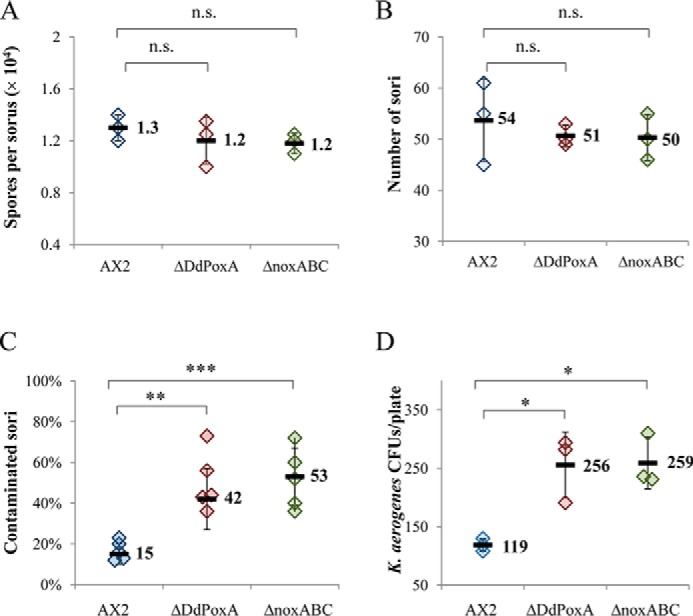
**Influence of DdPoxA on fruiting body sterility of *D. discoideum*.** *A* and *B*, comparison of *D. discoideum* wildtype (AX2), Δ*DdpoxA,* and Δ*noxABC* mutants regarding production of spores (*A*) and fruiting bodies (*B*). *Numbers in the graph* (*A*) refer to the average number of spores in one fruiting body. Significance of data was analyzed by using a *t* test with a *p* value cutoff of 0.05. *p* values are indicated as follows: *n.s.*, not significant (*p* > 0.05); *, *p* ≤ 0.05; **, *p* ≤ 0.01; ***, *p* ≤ 0.001. *Numbers in graph* (*B*) are counted fruiting bodies from drops of developed *D. discoideum* cells with an initial cell density of 1 × 10^6^ cells per drop. Both experiments were carried out in biological triplicates. *C,* contamination of fruiting bodies from *D. discoideum* wildtype (AX2), Δ*DdpoxA,* and Δ*noxABC*. Cells were grown on a lawn of *K. aerogenes*; cell development was started upon consumption of edible bacteria. *n* = 125 examined fruiting bodies for AX2 and Δ*DdpoxA* and 150 fruiting bodies for Δ*noxABC* in at least five biological replicates. *D,* number of colony-forming units of *K. aerogenes* in fruiting body suspensions from *D. discoideum* wildtype (AX2), Δ*DdpoxA,* and Δ*noxABC*. The experiment was carried out in biological triplicates.

Because DdPoxA expression was not detected in the vegetative phagocytosing amoebae, the initial hypothesis of its MPO-like function had to be revised. Subsequently, we focused on elucidating the putative roles of this peroxidase at the late development stages. We investigated the effects of the Δ*DdpoxA* knockout on the developmental cycle of *D. discoideum* in comparison with the wildtype AX2 and the Δ*noxABC* (*D. discoideum* knockout strain lacking the NoxA, -B, and -C, homologs of Nox2/gp91^Phox^, the catalytic subunit of NOX;) knockout strains. It has been shown in previous studies that the knockout of the three *nox* genes (each encoding a homolog of gp91^Phox^, the catalytic subunit of NADPH oxidases (NOX)) leads to a deficiency in immune defense at the slug and sorus stages (37, 38). NOXs are crucial enzymes producing reactive oxygen species during phagocytosis or pathogen attack (37). In contrast to DdPoxA, the NOXs are not secreted but are membrane-bound. RNAseq detection has shown that NoxB and NoxC are also up-regulated during cell development, whereas NoxA is predominantly expressed in the amoeboid stage (33, 34). To elucidate the impact of DdPoxA and the NOX enzymes during development, a defined concentration of *D. discoideum* cells (1 × 10^6^/ml) was plated on non-nutrient agar plates to induce starvation. Time-dependent morphological changes were documented using a stereoscope. Comparison of the Δ*DdpoxA* to the AX2 wildtype and the Δ*noxABC* mutant did not show any differences concerning cell development, indicating that DdPoxA was not essential for this process (Fig. S3). Furthermore, we demonstrated that there was no significant difference in the amount of produced fruiting bodies or spores between the three investigated variants (Fig. 7, *A* and *B*).

Finally, we probed the effect of the Δ*DdpoxA* knockout on the sterility of the sori in comparison with the AX2 wildtype and the Δ*noxABC* knockout mutant. For this, we cultivated *D. discoideum* cells on a lawn of *Klebsiella aerogenes* as a food source. After consumption of the bacteria, *D. discoideum* cells started the multicellular life cycle and formed fruiting bodies. Subsequently, we analyzed the sori of those fruiting bodies for the presence of live bacteria by plating them on fresh agar plates (Fig. 7*C*). After investigating a significant number of sori (*N* ≥ 125 for each mutant), we demonstrated that both Δ*DdpoxA* and Δ*noxABC*, showed a higher percentage of contaminated sori than the AX2 wildtype strain (43 and 52%, respectively, compared with 15% for the wildtype). Furthermore, we quantified the number of remaining bacteria in the sori. This was accomplished by resuspending a defined number of sori in Soerensen medium and plating the suspension on fresh selective agar plates. We found that sori from Δ*DdpoxA* and Δ*noxABC* strains contained twice as many contaminating bacteria as the wildtype AX2 (Fig. 7*D*). Hence, it was clearly shown that both mutants have significant deficiencies in keeping the slug and the sori sterile upon cell development.

## Discussion

Phylogenetic reconstruction of the main evolutionary lines of the mammalian heme peroxidases such as lactoperoxidase or myeloperoxidase revealed the presence of novel bacterial and early eukaryotic representatives within family 6 of the peroxidase–cyclooxygenase superfamily (7, 9). This work presents the first comprehensive biochemical study of an antibacterial peroxidase expressed in an organism that branched from the evolutionary tree close to the emergence of multicellularity. We focused on DdPoxA because *D. discoideum* is a social amoeba that can switch from a single-cell to a multicellular lifestyle under conditions of starvation. Moreover, DdPoxA is a typical representative for all related heme peroxidases from the whole genus *Dictyostelium* that already counts more than 80 distinct species (NCBI Taxonomy Database).

At the single cell stage, *D. discoideum* feeds on bacteria ingested by phagocytosis, a process that closely resembles bacterial killing within the neutrophil phagosome of vertebrates. In the absence of a food source, starting from single cell streams that chemotax toward a cAMP signal, a multicellular migrating slug is formed that finally differentiates into fruiting bodies containing persistent spores (5). At these late development stages, DdPoxA is shown to be up-regulated (Fig. 7*C*). These findings are supported by published RNAseq data on a slightly different *D. discoideum* laboratory strain (AX4) (33, 34).

Knockout of the *DdpoxA* gene had no observable impact on cell development, cell communication and aggregation, formation of the stalk, or the fruiting body. However, we showed that knockout of the *DdpoxA* gene significantly increased the bacterial contamination of the sori in comparison with the wildtype strain. This impact is very similar to the one already reported for a *D. discoideum* mutant lacking functional NOX (38, 39). These findings suggest that DdPoxA exhibits antibacterial activity that depends on H_2_O_2_ derived from dismutation of superoxide released by the NADPH oxidase(s).

Antibacterial activity requires DdPoxA secretion at the slug and fruiting body state as well as production of oxidants. A respective signal peptide translocates the nascent polypeptide chain to the ER. In the ER core glycosylation occurs; the heme group is inserted and, most probably, post-translationally modified as recently demonstrated for human promyeloperoxidase (40). Localization of GFP-tagged DdPoxA in the ER, the Golgi apparatus, and recycling endosomes clearly underlines that the likely glycosylated peroxidase follows the secretory pathway.

Similar to secreted mammalian peroxidases involved in antimicrobial activity (31, 41) DdPoxA exhibits a high thermal stability (*T_m_* >70 °C) in the pH range 5.0–7.0, which guarantees conformational stability under harsh conditions such as the one encountered in the soil environment of the amoeba.

Mammalian peroxidases and DdPoxA share a similar overall fold. The arrangement of the 21 α-helices and especially of the core helices, which provide the heme-binding ligands and catalytic residues, are fully conserved. Nevertheless, the number and localization of the disulfide bridges and the *N*-glycosylation sites, the nature of ligands of the distal Ca^2+^-binding site, as well as the length of the substrate channel are different between DdPoxA and mammalian peroxidases such as LPO.

As already demonstrated for chordata (family 1) peroxidases (18, 42), peroxidasin 1 (hsPxd01, family 2) (29), and a bacterial enzyme (family 6) (27, 28), the prosthetic group of DdPoxA is post-translationally modified by an autocatalytic radical mechanism that depends on hydrogen peroxide. However, DdPoxA is unique in having only one covalent ester bond between the hydroxymethyl group on the pyrrole ring A and Glu-236, whereas all other representatives studied so far have an additional ester bond between a conserved aspartate residue and the hydroxymethyl group of pyrrole ring C. In the case of DdPoxA, this acidic amino acid is replaced by Ile-100. All other amino acids of structural and functional relevance at the heme cavity are fully conserved in DdPoxA, including the proximal (His-325–Asn-403–Arg-406) and distal (Gln-97–His-101–Arg-233) triads. However, because of the lack of the second ester bond at pyrrole ring C, the stretch Gln-97–His-101 should be less constrained compared with LPO.

As a consequence, the spectral and redox properties of DdPoxA are different from family 1 proteins. The standard reduction potential of the Fe(III)/Fe(II) couple of DdPoxA is significantly more negative than that of LPO. Within the peroxidase–cyclooxygenase superfamily there is a clear correlation between the number of covalent ester bonds and *E*′_0_[Fe(III)/Fe(II)] values with *E*′_0_ (one ester bond, *e.g.* DdPoxA: −0.276 V) < *E*′_0_ (two ester bonds, *e.g.* LPO: −0.183 V; EPO: −0.176 V; LspPOX: −0.158 V; hsPxd01, −0.128 V) < *E*′_0_ (two ester bonds, one sulfonium ion linkage, MPO: +0.005 V) (27, 28, 43–45).

Closely related to the redox properties of these peroxidases is their capacity to oxidize halides by the redox intermediate compound I. Whereas the kinetics of the two-electron oxidation of the ferric peroxidases to compound I by hydrogen peroxide is typically very fast (3.2 × 10^6^ m^−1^ s^−1^ in case of DdPoxA) and does not depend on the post-translational modification of the prosthetic group (18, 30), the thermodynamics of the two-electron reduction of compound I by halides and thiocyanate strongly depend on the presence of heme to protein bonds. Only MPO is able to oxidize chloride (*E*′_0_ (HOCl/Cl^−^, H_2_O) = 1.28 V at pH 7.0) at reasonable rates (46, 47). Bromide oxidation (*E*′_0_ (HOBr/Br^−^, H_2_O) = 1.13 V at pH 7.0) has been demonstrated for EPO (48), LspPOX (27), and hsPxd01 (28). Oxidation of iodide (*E*′_0_ (HOI/I^−^, H_2_O) = 0.78 V at pH 7.0) and thiocyanate (*E*′_0_ (HOSCN/SCN^−^, H_2_O) = 0.56 V at pH 7.0) (46) is thermodynamically less challenging and thus typically is performed at high rates by compound I of all peroxidases of this superfamily. This applies also to DdPoxA that is unable to produce hypochlorous and hypobromous acid but oxidizes iodide and thiocyanate extremely efficiently (>10^8^ m^−1^ s^−1^).

This poses the question about the relation of this enzymatic activity and the role of DdPoxA in bacterial killing at the slug and fruiting body stage. It has been demonstrated by Klebanoff in 1967 (49) that human myeloperoxidase, iodide, and H_2_O_2_ have a bactericidal effect on *Escherichia coli* and that this effect corresponded to the iodination of the bacteria. Hypothiocyanous acid (HOSCN) is well known to be a potent antimicrobial species, being formed by LPO in human secretory mucosa, including the oral cavity, airway, and alimentary tract, thereby regulating the resident and transient flora as part of innate immunity (50). HOSCN is a weaker, more selective oxidant than HOCl or HOBr that reacts at biologically relevant rates with cysteine and/or selenocysteine residues of proteins and peptides and thus is responsible for arresting the spread of pathogens (51). Thus, the high rate of oxidation of iodide and thiocyanate by DdPoxA suggests a comparable antibacterial function in *D. discoideum* as described for mammalian peroxidases like LPO. Both thiocyanate and iodide are known to be found in the soil. Thiocyanate, for example, is known to be both secreted and utilized by various soil organisms as nitrogen source (52), whereas iodide is reported as the most prevalent form of iodine in soil and in rivers (53).

Summing up, in this report we present the first structural and functional characterization of a heme peroxidase with one heme to protein ester bond. Its high efficiency in H_2_O_2_-dependent iodide and thiocyanate oxidation and its expression at multicellular lifestyle of *D. discoideum* together with its antibacterial activity suggest that this secreted and highly stable glycoprotein acts in the first line defense of the fruiting bodies containing spores against bacteria.

## Experimental procedures

### Materials and reagents

Chemicals and enzymes were purchased from the following sources: the synthetic gene coding for DdPoxA and the expression vector were ordered from ATUM (Menlo Park, CA). Restriction enzymes and Endo H_f_ glucosidase were from New England Biolabs. Zeocin was purchased from Thermo Fisher Scientific. *P. pastoris* strain (BG11 with deleted AOX1 ORF) was purchased from bisy e.U. (Hofstätten, Austria). Chelating Sepharose fast flow column was from Amersham Biosciences; SEC column (HiLoad 16/600 Superdex 200 pg, preparation grade) was purchased from GE Healthcare. Centriprep 30 was from Amicon; PD10 desalting columns were from Sigma, and Whatman filter papers were from GE Healthcare. Polyclonal antibodies against the recombinant DdPoxA raised in rabbits and purified by protein A-Sepharose chromatography were ordered from Seramun Diagnostica GmbH (Heidesee, Germany). All other chemicals were purchased from Sigma at highest available grade.

### Cloning, expression, and purification

The gene coding for DdPoxA (Uniprot accession code Q6TMK4) was synthesized with an additional N-terminal His_6_ tag and codon-optimized for expression in *P. pastoris* at DNA 2.0. The signal peptide prediction server SignalP 4.1 (54) was used to identify the secretion signal, which was subsequently removed. The gene was cloned into the pJ912 shuttle vector carrying a Zeocin resistance and the α-factor from *Saccharomyces cerevisiae* as signal sequence for secretion into the extracellular space. The plasmid was transferred into electrocompetent *E. coli* Top 10 cells for amplification. The purified plasmid was linearized using the restriction enzyme SwaI and transformed into the *P. pastoris* BG11 expression strain by electroporation (Bio-Rad electroporator with VWR cuvettes, option “Fungi Sc2”). Transformants were selected on YPD plates (10 g/liter yeast extract, 20 g/liter peptone, 10 g/liter glucose, 15 g/liter agar) supplemented with Zeocin (100 mg/liter). The transformed *P. pastoris* BG11 cells were initially cultivated in YP medium supplemented with 1% glycerol at 28 °C and 180 rpm overnight. Subsequently, 10-ml aliquots of these precultures were further used to inoculate 200 ml of fresh YP-glycerol medium supplemented with biotin solution (final concentration 4 mg/liter) in baffled shaken flasks and incubated at 28 °C and 180 rpm. After complete glycerol consumption (∼24 h), protein expression was induced by adding methanol to 0.5% final concentration. Expression proceeded for further 24 h at 25 °C and 180 rpm. Subsequently, methanol was added to 1% final concentration. Hemin solution (10 μm final concentration at pH 9–10) was added shortly after addition of methanol, and incubation was continued. After 24 h, methanol was added once more to a 1% final concentration. Four hours after last addition of methanol, the supernatant was separated from the cells by centrifugation (3000 × *g*, 10 min, 4 °C). The supernatant was stored at −30 °C until further purification.

Recombinant DdPoxA was purified from the *P. pastoris* cell supernatant by metal chelate affinity chromatography using the N-terminal His_6_ tag. First, proteins were precipitated using ammonium sulfate in two steps. In the first step, 170 g/liter ammonium sulfate (31% saturation) was added stepwise at 4 °C to the cell supernatant. Subsequent centrifugation (45,000 × *g*, 20 min, 4 °C) led to precipitation of host proteins. In the second step, 343 g/liter ammonium sulfate (82% final saturation) was added stepwise at 4 °C to the remaining supernatant, and the recombinant protein was precipitated by centrifugation (45,000 × *g*, 20 min, 4 °C). The pellet was dissolved in binding buffer (67 mm phosphate buffer supplemented with 20 mm imidazole and 0.5 m NaCl (pH 7.2), 50 ml/liter of *P. pastoris* cell culture) for subsequent purification by metal chelate affinity chromatography. The column was loaded with Ni^2+^ ions and equilibrated with 3 column volumes of binding buffer. The protein solution was loaded on the column, followed by extensive washing (6 column volumes) with binding buffer. A linear gradient from 20 to 250 mm imidazole was used for elution, and 1.5-ml fractions were collected. The fractions were investigated by SDS-PAGE and UV-visible spectroscopy, and the purest fractions were concentrated (Amicon centrifugal filters, 30-kDa cutoff), desalted using PD10 columns, and stored in 10 mm phosphate buffer (pH 7.0), at −80 °C.

### UV-visible and electronic circular dichroism spectroscopy

UV-visible spectra were recorded using a Hitachi U-3900 spectrophotometer from 200 to 800 nm at 25 °C in 10 mm phosphate buffer (pH 7.0). The molar extinction coefficient of DdPoxA has been determined by the method of Bradford and shows a value of 84,950 m^−1^ cm^−1^ at 416 nm (55). It was used for all calculations of protein concentrations of DdPoxA.

Electronic circular dichroism (ECD) measurements were performed with Chirascan (Applied Photophysics) that allowed simultaneous measurement of UV-visible and ECD spectra at defined temperature using a Peltier temperature control unit. The machine was flushed with nitrogen with a flow rate of 5 liters/min before and throughout the measurements. For probing the overall secondary structure composition in the far-UV region (180–260 nm), the path length of the cuvette was 1 mm; bandwidth was 3 nm, and scan time was 10 s per point. Concentration of DdPoxA was 0.5 mg/ml in 10 mm phosphate buffer (pH 7.0). For unfolding studies, changes in the ellipticity at 208 nm were followed between 25 and 95 °C (1 °C increase per min). To obtain information about tertiary structure and heme insertion, the ECD spectrum at 25 °C was recorded in the near-UV and visible region (250–470 nm) with the path length and spectral bandwidth set to 10 and 1 nm, respectively. For studying unfolding of the heme cavity, the ellipticity was monitored at the Soret minimum at 408 nm between 25 and 95 °C (1 °C increase per min).

### Size-exclusion chromatography combined with multiangle light scattering

Protein purity and the oligomeric state of the recombinantly produced DdPoxA were determined by size-exclusion chromatography (SEC) combined with MALS. Measurements were performed on an LC20 prominence HPLC system equipped with the refractive index detector RID-10A, the photodiode array detector SPD-M20A (all from Shimadzu), and a MALS Heleos Dawn8+ plus QELS detector (Wyatt Technology). The column (Superdex 200 10/300 GL, GE Healthcare) was equilibrated with Dulbecco's PBS plus 200 mm NaCl (pH 7.2) as running buffer. Experiments were carried out at a flow rate of 0.75 ml/min at 25 °C and analyzed using the ASTRA 6 software (Wyatt Technology). Accuracy of molar mass determination was verified by measuring a sample containing bovine serum albumin. The protein (25 μg per analysis) was centrifuged (17,000 × *g*, 10 min, 20 °C) and filtered (0.1 μm Ultrafree-MC filter from Merck Millipore) before applying to the column.

### Spectroelectrochemistry

The standard reduction potential *E*′^0^ of the Fe(III)/Fe(II) couple of the heme protein was measured using a homemade OTTLE cell (optically transparent thin layer spectroelectrochemical cell) in a three-electrode configuration. The setup was composed of a gold mini-grid working electrode (Buckbee-Mears), an Ag/AgCl/KCl_sat_ micro-reference electrode, separated from the working solution by a Vycor set, and a platinum wire as counter-electrode. The reference electrode was calibrated against a saturated calomel electrode (Hg_2_Cl_2_) before each set of measurements. The potentials were applied using a potentiostat/galvanostat (Amel model 2053). UV-visible spectra were recorded using a Varian Cary C50 spectrophotometer, flushed with argon. The obtained potentials were referenced to the standard hydrogen electrode. Spectroelectrochemical titrations were carried out using samples containing 10 μm DdPoxA in 100 mm phosphate buffer (pH 7.0) and 100 mm NaCl, at 25 °C. 100 μm methyl viologen and 2 μm lumiflavin 3-acetate, methylene blue, phenazine methosulfate, and indigo disulfonate were used as redox mediators.

### Differential scanning calorimetry

DSC measurements were conducted using a VP-DSC microcalorimeter (MicroCal) controlled by the VP-viewer program and equipped with a 137-μl cell and an autosampler for 96-well plates. Samples were measured with a concentration of 10 μm in 50 mm phosphate buffer (pH 7.0). The heating scan rate was 60 °C/h, and the cell pressure was constant at 4.1 bar; the temperature range was programmed from 20 to 100 °C. Data analysis was performed using the MicroCal Origin7 software. Raw DSC data were baseline-corrected with buffer and normalized for the applied protein concentration. The resulting endotherms were fitted by a non-two-state transition model.

### SDS-PAGE and enhanced chemiluminescence (ECL)

Protein separation by SDS-PAGE was carried out using Mini Protean TGX gels (Bio-Rad) in Tris/glycine/SDS running buffer (Bio-Rad) at 250 V. The gels were stained with Coomassie Brilliant Blue staining solution with Precision Plus Protein (All Blue) as the prestained ladder (Bio-Rad). The iBlot Dry Blotting System (Invitrogen) with nitrocellulose membranes was used for immobilizing proteins on nitrocellulose membranes (iBlot Gel Transfer Stacks, nitrocellulose mini). Enhanced chemiluminescence was performed using the Bio-Rad ChemiDoc XRS+ Imaging System. The blotted membrane was incubated with Clarity Western ECL Substrate, applying an exposure time between 1 and 20 s (depending on sample concentration). Note that non-covalently bound heme is lost during gel electrophoresis. The ECL detection reagent relies on the hydrogen peroxide-mediated oxidation of luminol to the light-emitting 3-aminophthalate in the presence of a catalyst like heme.

### Mass spectrometry

Prior to mass spectrometric analysis (MS), deglycosylation of purified DdPoxA was conducted using the glucosidase Endo Hf overnight at 37 °C and 180 rpm. Deglycosylated protein was further purified by SEC on a HiLoad 16/600 Superdex 200 pg (preparation grade) column, equilibrated with 50 mm phosphate buffer (pH 7.0). The eluted protein solution was concentrated and stored at −80 °C.

To detect the mass of the deglycosylated protein, intact protein mass spectrometry analysis was performed. In detail, 2.5 μg of deglycosylated DdPoxA were analyzed using a Dionex Ultimate 3000 system directly linked to a QTOF instrument (maXis 4G ETD, Bruker) equipped with the standard ESI source in the positive ion mode. Data were recorded within a range from 400 to 3800 *m*/*z*. The instrument was calibrated using the ESI calibration mixture (Agilent). For protein separation, a ProSwift^TM^ RP-4H analytical separation column (Thermo Fisher Scientific) (250 × 0.200 mm) was used. A gradient from 80% solvent A and 20% solvent B (solvent A, 0.05% trifluoroacetic acid (TFA); solvent B, 80.00% acetonitrile, 19.95% H_2_O, and 0.05% TFA) to 65% B in 20 min was applied, followed by a 15-min gradient from 65 to 95% B, at a flow rate of 8 μl/min at 65 °C. The obtained data were processed using Data Analysis 4.0 (Bruker), and the obtained spectrum was deconvoluted by MaxEnt (Maximum Entropy Method, low mass, 40,000; high mass, 200,000, instrument resolving power 100,000).

### Crystallization, X-ray data collection, structure determination, and refinement

Crystallization experiments were performed using the sitting drop vapor diffusion method in SWISSCI MRC three-well crystallization plates (Molecular Dimensions, Newmarket, UK). Crystallization drops were set using a mosquito crystallization robot (TTP Labtech, UK). The reservoir was filled with 40 μl of precipitant solution. In the sample wells, ratios of 100:150, 150:150, and 200:150 nl of protein to precipitant were dispensed. Protein concentration was 10 mg/ml in 10 mm phosphate buffer (pH 7.0). Commercially available crystallization screens were used for initial screening. Crystallization plates were stored in a Formulatrix RI-1000 imaging device at 22 °C. Successful hits were obtained using the JCSG-plus^TM^ from Molecular Dimensions. Initial screening conditions were optimized for growth of larger crystals, yielding final conditions as follows: 0.2 m BisTris (pH 5.5), 0.2 m MgSO_4_, 23.2% PEG 3350 (Table 1).

**Table 1:** **Data collection and refinement statistics**

	DdPoxA (6ERC)
**Data collection**	
Wavelength (nm)	1.072
Resolution range (Å)	48.67–2.5 (2.589–2.5)*^a^*
Space group	P 3_1_ 2 1
Unit cell (Å)	128.253, 128.253, 146.015, 90, 90, 120
Total reflections	485,020 (46,671)
Unique reflections	48,501 (4813)
Multiplicity	10.0 (9.7)
Completeness (%)	99.90 (99.98)
Mean *I*/σ(*I*)	7.64 (0.60)
Wilson *B*-factor (Å^2^)	55.76

**Refinement**	
*R*-merge (%)	0.3149 (4.14)
*R*-meas (%)	0.3319 (4.372)
*R*-pim (%)	0.1043 (1.395)
*CC*½	0.995 (0.298)
CC*	0.999 (0.678)
Reflections used in refinement	48462 (4812)
Reflections used for *R*-free	1189 (113)
*R*-work	0.2311 (0.3814)
*R*-free	0.2594 (0.3385)
*CC* (work)	0.954 (0.527)
*CC* (free)	0.928 (0.440)
No. of non-hydrogen atoms	8586
No. of atoms macromolecules	8240
No. of atoms ligands	182
No. of atoms solvent	164
Protein residues	1022
r.m.s. (bonds) (Å)	0.005
r.m.s. (angles) (°)	0.8
Ramachandran favored (%)	96.37
Ramachandran allowed (%)	3.63
Ramachandran outliers (%)	0
Rotamer outliers (%)	0.11
Clashscore	1.51
Average *B*-factor (Å^2^)	64.84
Macromolecules (Å^2^)	65.41
Ligands (Å^2^)	54.96
Solvent (Å^2^)	47.02
No. of TLS groups	2

*^a^* Values in parentheses represent the highest resolution shell.

The crystal was soaked with mother liquor supplemented with 20% 2-methyl-2,4-pentanediol, harvested using a cryo-loop, and flash-vitrified in liquid nitrogen. Datasets were collected at beam-line ID29 (56) of European Synchrotron Radiation Facility (ESRF, Grenoble, France) at 100 K using a DECTRIS PILATUS 6M detector.

The dataset was processed with XDS, and symmetry equivalent reflections were merged with XDSCONV (57). Intensities were not converted to amplitudes. Initially, the high-resolution cutoff was 2.31 Å (*CC*_½_ = 0.23) (58). The phase problem was solved by molecular replacement using balbes (29). The model was further improved by iterative cycles of manual model building using COOT (59) and maximum likelihood refinement using PHENIX-Refine (60). PHENIX-Refine converted intensities into amplitudes using the French and Wilson algorithm (61). The final high-resolution cutoff was based on performing paired refinement using the PDB_REDO web server (62). Final stages of refinement included Translation Liberation Screw (TLS) parameters, isotropic B-factor model, automated addition of hydrogens and water molecules, optimization of X-ray/ADP weight, and optimization of X-ray/stereochemistry weight. The model was validated with MolProbity (63). Figures were prepared with PyMOL Molecular Graphics System (Version 1.3, Schrödinger, LLC). Atomic coordinates have been deposited in the Protein Data Bank under accession code 6ERC. OMIT maps were calculated by removing the heme followed by refinement in phenix.refine (59). FEM maps were calculated using the feature-enhanced map tool in PHENIX (59).

The r.m.s.d. values and *Z*-scores were calculated using the PDBe Fold version 2.59 server with a lowest acceptable match of 70% (64). CAVER (65) was used to detect putative substrate channels of DdPoxA and goat LPO (PDB code 2R5L). For calculation of the characteristics of the channels, the heme iron was set as a starting point for both proteins. Channels were calculated with the following settings: minimum probe radius, 0.9 Å; shell depth, 4 Å; shell radius, 3 Å; clustering threshold, 3.5; number of approximating balls, 12; input atoms: 20 amino acids.

### Stopped-flow spectroscopy

Pre-steady-state kinetic experiments were performed to study the relevant redox intermediates of DdPoxA. The experiments were carried out with a stopped-flow apparatus (model SX-18MV and Pi-star-180, Applied Photophysics) in the conventional or sequential mode. The optical quartz cell with a path length of 10 mm had a volume of 20 μl. The dead time of both stopped-flow machines was 1.0 ms. The spectra were followed with the photodiode array detector, and the single wavelength time traces were recorded using a photomultiplier detector (Applied Photophysics). All measurements were performed at 25 °C in triplicates.

Binding of cyanide and reaction with hydrogen peroxide were performed in the conventional stopped-flow mode. The first syringe contained 2–4 μm protein solution in 50 mm phosphate buffer (pH 7.0), and the second syringe contained cyanide or hydrogen peroxide solutions in varying concentrations. Rates of cyanide binding and compound I formation were obtained by single or double exponential fitting of the time traces at 412 nm. Pseudo-first-order rate constants, *k*_obs_, were used to calculate the apparent bimolecular binding constant (*k*_on_) by plotting *k*_obs_ values *versus* cyanide concentration. From the *x*-intercept of this plot, *k*_off_, the dissociation rate constant, was estimated, enabling calculation of the dissociation constant *K_D_* = *k*_off_/*k*_on_.

For determination of compound I formation, the obtained pseudo-first-order rate constants (*k*_obs_) were used to calculate the apparent bimolecular rate constant (*k*_app_) from the slope of the plot of *k*_obs_ values *versus* hydrogen peroxide concentration. Multi-mixing, sequential stopped-flow spectroscopy was performed to monitor the reduction of DdPoxA compound I using the two-electron donors Cl^−^, Br^−^, I^−^, and SCN^−^. A solution containing 4–8 μm DdPoxA was premixed with a 2.5 m excess of hydrogen peroxide in the aging loop for 400 ms to form compound I.

### D. discoideum and K. aerogenes cell culture

The *D. discoideum* laboratory strain AX2 and the mutant strains Δ*DdpoxA* and Δ*noxABC* (38) were cultivated axenically in 10-cm Petri dishes in HL5c medium (5 g/liter peptone, 5 g/liter thiotone E peptone, 10 g/liter glucose, 5 g/liter yeast extract, 0.35 g/liter Na_2_HPO_4_·7H_2_O, 0.35 g/liter KH_2_PO_4_, 0.05 g of dihydrostreptomycin-sulfate (pH 6.5)) supplemented with 50 units/ml penicillin and 50 mg/ml streptomycin (Pen/Strep) at 22 °C. The exponentially growing cells were harvested at about 80% confluence for experiments. To obtain higher cell numbers, *D. discoideum* was transferred into shaking flasks with HL5c medium (plus Pen/Strep) at 22 °C, and cells were harvested with a maximal density of 5 × 10^6^/ml. The avirulent laboratory wildtype strain of *K. aerogenes* was cultured overnight in LB medium (10 g/liter peptone, 5 g/liter yeast extract, 5 g/liter NaCl (pH 7.0)) without antibiotics at 37 °C and 180 rpm.

### Expression of DdPoxA during cell development

*D. discoideum* cells were grown in shaking culture in HL5c medium (+ Pen/Strep), harvested by centrifugation (1600 rpm, 4 °C, 4 min), and washed three times by resuspending the pellet in 0.5 volume of cold sterile development buffer (5 mm Na_2_HPO_4_, 5 mm KH_2_PO_4_, 1 mm CaCl_2_, 2 mm MgCl_2_). For each required time point, 2 × 10^8^ cells were resuspended in 2 ml of cold development buffer, and 1 aliquot was frozen as time point 0. For each time point, one 10-cm Petri dish was prepared with three Whatman No. 3 filter papers and one Whatman No. 50 on top. The filter papers were soaked with 5 ml of development buffer; the remaining air bubbles were removed with a sterile spreader, and any excess liquid was aspirated. The cell suspensions were slowly distributed over the filter. Cells were incubated at 22 °C in a humid box. For cell harvesting, the no. 50 filter paper was placed in a Falcon tube; 20 ml of cold development buffer was added and mixed to remove cells from the filter. The cell suspension was centrifuged (1600 rpm, 4 °C, 4 min) and frozen (66). Subsequently, *D. discoideum* cells were thawed by immediately adding 400 μl of lysis buffer to the pellet. Cell lysis was carried out by sonication (micro tip, 3 × 20 s per sample, 50% duty cycle), and cell debris was centrifuged (13,000 rpm, 4 °C, 15 min). Protein concentration was determined by Nanodrop using the formula for DNA-contaminated protein samples: *c*(mg/ml) = 1.55 × (*A*_280_) − 0.76 × (*A*_260_). Samples were diluted to a concentration of 10 mg/ml in hot SDS-PAGE sample buffer for gel electrophoresis. Protein separation by SDS-PAGE was carried out using 8% acrylamide gels at 150 V. The separated proteins were blotted on nitrocellulose membranes (120 V, 4 °C, 90 min) and stained with Ponceau S to verify that the total amount of protein is constant in all samples. After washing the membrane with PBS-T, the blotted proteins were blocked with Blotting-Grade Blocker (Bio-Rad) and detected with an anti-DdPoxA as primary antibody and an anti-rabbit antibody conjugated to HRP as secondary antibody. As a positive control, the blot was stripped and incubated again with anti-actin antibody as primary antibody and anti-mouse conjugated with HRP as secondary antibody. Band development was carried out with the Clarity Western ECL substrate (Bio-Rad).

### Comparative development of D. discoideum

*D. discoideum* wildtype (AX2), ΔDdPoxA, and ΔnoxABC were grown in HL5c medium (+ Pen/Strep) to a maximal cell density of 5 × 10^6^/ml. Cells were centrifuged (1600 rpm, 4 °C, 4 min) and washed three times with Soerensen medium (2 g/liter KH_2_PO_4_, 0.29 g/liter Na_2_HPO_4_ (pH 6.0)). Drops of *D. discoideum* cells with a known concentration (1 × 10^6^/ml) were plated on Soerensen agar plates (2 g/liter KH_2_PO_4_, 0.29 g/liter Na_2_HPO_4_ (pH 6.0), 15 g/liter agar) and incubated at 22 °C in a humid box. Pictures were taken with a stereoscope at desired time points during development.

### Investigation of fruiting body sterility

0.2 ml of overnight culture of *K. aerogenes* in LB medium was mixed with 8 × 10^4^ *D. discoideum* cells. The cell mixture was plated on SM/2 agar plates (5 g/liter glucose, 5 g/liter bacto peptone, 0.5 g/liter yeast extract, 1 g/liter MgSO_4_ × 7H_2_O, 1 g/liter Na_2_HPO4, 2.2 g/liter KH_2_PO4, 15 g/liter agar) and incubated 3–4 days at 22 °C upside down in a humid box, until *D. discoideum* cells have formed fruiting bodies. To investigate the contamination of individual fruiting bodies, single sori were picked up carefully with sterile pipette tips and transferred to a new SM/2 agar plate. The plates were incubated overnight at 37 °C (a temperature that fully restricts *D. discoideum* growth) to grow residual *K. aerogenes* cells, and the number of contaminated fruiting bodies was counted. To quantify the number of residual bacteria, 25 fruiting bodies were taken up with sterile pipette tips and dissolved in 500 μl of Soerensen medium. 50 μl of the suspension were plated on new SM/2 agar plates. The plates were incubated at 37 °C overnight to grow *K. aerogenes* cells, and the number of bacterial colonies was counted. The experiment was carried out in biological triplicate.

### Determination of number of spores and fruiting bodies

10 fruiting bodies of each *D. discoideum* wildtype (AX2) and the mutants Δ*DdpoxA* and Δ*noxABC* were randomly picked and resuspended in 100 μl of Soerensen medium. The number of spores in the solution was determined using a hemocytometer. The number of produced fruiting bodies was determined by counting fruiting bodies in the comparative development experiment (see above).

### Cloning, expression, and localization of DdPoxA-GFP

The *poxA* CDS was PCR-amplified with forward primer poxA-FL-F (tttagatctaaaaATGCGATTAAATTTAATATCGTTTTTTATAATATTAC), which contains a BglII site, and reverse primer poxA-FL-R (tttactagtTTTTCTAAAAACATTTGGTTGAACATAACCAATAT), which contains an SpeI site, from cDNA generated from *D. discoideum* cells undergoing development. The PCR product was cloned into pJET1.2 using the CloneJET PCR cloning kit (Thermo Fisher Scientific) and sequenced. The *poxA*-containing fragment was excised by sequential digestion with SpeI and BglII, gel-extracted, and ligated into pDM323 (67), also cut with SpeI and BglII, upstream of *gfp* to create a *poxA-gfp* fusion. Amoeba stage *D. discoideum* cells were transfected with the resulting plasmid and selected with 5 μg/ml G418. PoxA-GFP-expressing cells were seeded on glass coverslips and fixed in ultracold methanol (68). After blocking with PBS, 0.3% gelatin, cells were stained with rabbit anti-GFP (MBL), a mixture of four mouse monoclonal antibodies recognizing PDI (21), rat anti-rabbit antibody labeled with AlexaFluor 488 (Thermo Fisher Scientific), and rat anti-mouse antibody labeled with AlexaFluor 594 (Thermo Fisher Scientific). Nuclei were stained with DAPI, and coverslips were mounted on microscope slides with ProLong Gold antifade reagent (Thermo Fisher Scientific). Images were taken with a Axio Imager Z1m (Zeiss) and processed using ImageJ.

## Author contributions

A. N., J. D. D., and G. M. formal analysis; A. N., J. D. D., G. M., and M. B. investigation; A. N. and J. D. D. visualization; A. N. and C. O. writing-original draft; J. D. D., M. B., G. B., P. G. F., T. S., and C. O. supervision; J. D. D., G. M., M. B., M. Z., G. B., P. G. F., T. S., and C. O. methodology; J. D. D., G. M., M. Z., G. B., K. D.-C., P. G. F., and T. S. writing-review and editing; G. M., G. B., K. D.-C., T. S., and C. O. resources; G. M. and P. G. F. software; G. B. and T. S. validation; P. G. F., T. S., and C. O. conceptualization; C. O. funding acquisition.

## Supplementary Material

Supporting Information

## Footnotes

4 The abbreviations used are:

LPO: lactoperoxidase
AX2: *D. discoideum* wildtype laboratory strain
DdPoxA: peroxidase from *D. discoideum*
DSC: differential scanning calorimetry
ECD: electronic circular dichroism
EPO: eosinophil peroxidase
hsPxd01: human peroxidasin 1
HOX: hypohalous acid
LspPOX: peroxidase from the cyanobacterium *Lyngbya* sp. PCC 8106
MALS: multiangle light scattering
MPO: myeloperoxidase
ER: endoplasmic reticulum
NOX: NADPH oxidase
SEC: size-exclusion chromatography
MALS: multiangle light-scattering
r.m.s.d.: root mean square deviation
Endo H_f_: endo-β-*N*-acetylglucosaminidase
PDI: protein-disulfide isomerase
PDB: Protein Data Bank
BisTris: 2-[bis(2-hydroxyethyl)amino]-2-(hydroxymethyl)propane-1,3-diol
RNAseq: RNA sequenced
Pen/Strep: penicillin/streptomycin
ECL: enhanced chemiluminescence
ESI: electrospray ionization.
