# Supplementary material for: Secreted heme peroxidase from Dictyostelium discoideum: Insights into catalysis, structure, and biological role
Source: J Biol Chem. 2017 Dec 14;293(4):1330–45. doi: 10.1074/jbc.RA117.000463 (PMC5787809; doi:10.1074/jbc.RA117.000463)
Supplement: Supporting Information [file supp_293_4_1330__index.html]

Secreted Heme Peroxidase from Dictyostelium discoideum: Insights into Catalysis, Structure and Biological Role — Secreted heme peroxidase from Dictyostelium discoideum — Secreted heme peroxidase from Dictyostelium discoideum: Insights into catalysis, structure, and biological role — Secreted heme peroxidase from Dictyostelium discoideum — Supporting Information 

# Secreted heme peroxidase from *Dictyostelium discoideum*: Insights into catalysis, structure, and biological role

## Supporting Information

- Supplemental Information - Supplemental Figures S1 - S3
