# Supplementary material for: Secreted heme peroxidase from Dictyostelium discoideum: Insights into catalysis, structure, and biological role
Source: J Biol Chem. 2017 Dec 14;293(4):1330–45. doi: 10.1074/jbc.RA117.000463 (PMC5787809; doi:10.1074/jbc.RA117.000463)
Supplement: Supporting Information [file supp_RA117.000463_133183_1_supp_31172_p0y0t5.pdf]

SUPPLEMENTAL INFORMATION

Secreted Heme Peroxidase from *Dictyostelium discoideum*: Insights into Catalysis, Structure and Biological Role

Andrea Nicolussi[1], Joe Dan Dunn[2], Georg Mlynek[3], Marzia Bellei[4], Marcel Zamocky[1,5], Gianantonio Battistuzzi[6], Kristina Djinović-Carugo[3,7], Paul G. Furtmüller[1], Thierry Soldati[2], and Christian Obinger[1] *

[1] Department of Chemistry, Division of Biochemistry, BOKU - University of Natural Resources and Life Sciences, 1190 Vienna, Austria; [2] Department of Biochemistry, Faculty of Science, University of Geneva, 1211 Genève, Switzerland; [3] Department for Structural and Computational Biology, Max F. Perutz Laboratories, University of Vienna, 1030 Vienna, Austria; [4] Department of Life Sciences, University of Modena and Reggio Emilia, via Campi 103, 41125 Modena, Italy; [5] Institute of Molecular Biology, Slovak Academy of Sciences, 84551 Bratislava, Slovakia;
[6] Department of Chemistry and Geology, University of Modena and Reggio Emilia, 41125 Modena, Italy; [7] Department of Biochemistry, Faculty of Chemistry and Chemical Technology, University of Ljubljana, 1000 Ljubljana, Slovenia

Running title: Secreted heme peroxidase from *Dictyostelium discoideum*

*Author to whom correspondence should be addressed: Christian Obinger. Phone: +43-1-47654-77273; fax: +43-1-47654-77059, email: christian.obinger@boku.ac.at

```
DdPoxA       --------------------------------------------------------------------------------   0
goat LPO     --------------------------MLVCLHLQVFLASVALFEV--AASDTIAQAASTTTISDAVSKVKTQVNKAFLDSRTRLKTALSS  62
human EPO    --------------------------MHLLPALAGVLATLVLAQPCEGTDPASPGAVETSVLRDCIAEAKLLVDAAYNWTQKSIKQRLRS  64
human MPO    MGVPFFSSLRCMVDLGPCWAGGLTAEMKLLLALAGLLAILATPQPSEGAAPAVLGEVDTSLVLSSMEEAKQLVDKAYKERRESIKQRLRS  90

DdPoxA       ---------------------------------------------------------MRLNLISFF---IIFTILVSISNSQEFRSYTGE  30
goat LPO     EAPTTRQLSEYFKHAKGRTRTAIRNGQVWEESLKRLRRD-------T-TLTNVTDPSLELTALSWEVGCGAPVPLVTCDEQSPYRTITGD 144
human EPO    GSASPMDLLSYFKQPVAATRTVVRAADYMHVALGLLEEKLQPQRSGPFNVTDVL-TEPQLRLLSQASGCALRDQAERCS--DKYRTITGR 151
human MPO    GSASPMELLSYFKQPVAATRTAVRAADYLHVALDLLERKLRSLWRRPFNVTDVL-TPAQLNVLSKSSGCAYQDVGVTCPEQDKYRTITGM 179
                                                                       .*  :*              . :*: **

                         S1                                   H1                       H2
DdPoxA       GNNKQNPKQGSIFTPFIRLAN---------PI----KFNKNGFPNITNQPSRAISNIIFDQQT-HIGSKEHLTDMFNMWGQFLIHNMALS 106
goat LPO     CNNRRSPALGAANRALARWLPAEYEDGLAVPFGWTQRKTRNGFRVP---LAREVSNKIVGYLDEEGVLDQNRSLLFMQWGQIVDHDLDFA 231
human EPO    CNNKRRPLLGASNQALARWLPAEYEDGLSLPFGWTPSRRRNGFLLP---LVRAVSNQIVRFPNERLTSDRGRALMFMQWGQFIDHDLDFS 238
human MPO    CNNRRSPTLGASNRAFVRWLPAEYEDGFSLPYGWTPGVKRNGFPVA---LARAVSNEIVRFPTDQLTPDQERSLMFMQWGQLLDHDLDFT 266
              **:: *  *:    : *           *        :***      * :** *.     .   ..  : :*  ***:: *:: ::

                                                              S2                S3         H3
DdPoxA       KPE-----------------PNSWPIKVPKCDQYFDPACIGNKTMNYFRTRATEVPCDVGKTVVDEDGKCYEQINSLGSYIDGNVLYG 177
goat LPO     PETELGSSEHSKVQCEEYCVQGDECFPIMFPKNDPKLKT---QGKCMPFFRAGFV---CPTP----PYQSLARDQINAVTSFLDASLVYG 311
human EPO    PESPARVAFTAGVDCERTCAQLPPCFPIKIPPNDPRIKN---QRDCIPFFRSAPS---CPQN----K--NRVRNQINALTSFVDASMVYG 316
human MPO    PEPAARASFVTGVNCETSCVQQPPCFPLKIPPNDPRIKN---QADCIPFFRSCPA---CPGS----N--ITIRNQINALTSFVDASMVYG 344
                                  .:*: .*  *  :.      . : :**:      *               :***:: *::*..::**

                H4                                          H5                    H6
DdPoxA       NSEEICKNLRSLSGGEMKMTVTDV----G--DLPPKNVPGVPMDNDANLFPIDQLYSVGERRGNENPGLLSIHTLLLRDHNRLARKFARL 261
goat LPO     SEPSLASRLRNLSSPLGLMAVNQEAWDHGLAYPPFNNVKPSPCEF-INTTAHVPCFQAGDSRASEQILLATVHTLLLREHNRLARELKRL 400
human EPO    SEVSLSLRLRNRTNYLGLLAINQRFQDNGRALLPFDNLHDDPCLL-TNRSARIPCFLAGDTRSTETPKLAAMHTLFMREHNRLATELRRL 405
human MPO    SEEPLARNLRNMSNQLGLLAVNQRFQDNGRALLPFDNLHDDPCLL-TNRSARIPCFLAGDTRSSEMPELTSMHTLLLREHNRLATELKSL 433
             ..  :. .**. :.    ::::.:     *    * .*:   *    *       : .*:*..*   * ::***::*:***** ::

                                H7                            H8
DdPoxA       HPEWDDERVFQQSRSCIIEQIQKITYDEYLPTTLGS------FPSYTGYDANVNAQVSNEFTTTAFRFGHSEVGPFMEYYSENGTRLQP- 344
goat LPO     NPHWDGEMLYQEARKILGAFIQIITFRDYLPIVLGS-EMQKWIPPYQGYNNSVDPRISNVFT-FAFRFGHMEVPSTVSRLDENYQPWGPE 488
human EPO    NPRWNGDKLYNEARKIMGAMVQIITYRDFLPLVLGKARARRTLGHYRGYCSNVDPRVANVFT-LAFRFGHTMLQPFMFRLDSQYRASAPN 494
human MPO    NPRWDGERLYQEARKIVGAMVQIITYRDYLPLVLGPTAMRKYLPTYRSYNDSVDPRIANVFT-NAFRYGHTLIQPFMFRLDNRYQPMEPN 522
             * :*.*:.: :::::*. :   :* **:::**.**      :  * .*  .*: :::* **  ***:**  :   :   ...     *

                H9     H10       H11              H12                 H13         H14
DdPoxA       --LPIKFSYFNP--HALNRGVEPLIRGLIINEEE--NIDIYMISDLRNFLFGKPGQ-GGLDLASRNLQRNRDHGIPPYNSLRRQLGLRPV 427
goat LPO     AELPLHTLFFNTWRIIKDGGIDPLVRGLLAKNSKLMNQNKMVTSELRNKLFQPTHKVHGFDLAAINLQRCRDHGMPGYNSWRGFCGLSQP 578
human EPO    SHVPLSSAFFASWRIVYEGGIDPILRGLMATPAKLNRQDAMLVDELRDRLFRQVRR-IGLDLAALNMQRSRDHGLPGYNAWRRFCGLSQP 583
human MPO    PRVPLSRVFFASWRVVLEGGIDPILRGLMATPAKLNRQNQIAVDEIRERLFEQVMR-IGLDLPALNMQRSRDHGLPGYNAWRRFCGLPQP 611
               :*:   :*      : *::*::***: .  :  . :     .::*: **    :  *:** : *:** ****:* **: *   **

                H15     H16       H17    H18                 H19               H20
DdPoxA       QTWS---DITSDPQIQNRLKNAYKSVDDIDSYVGGLAEDHMEGSCVGQTFYLIIYEQFFRTRAGDRFWYETPEMRMVNREC--ETTTFAE 512
goat LPO     KTLKGLQAVLKNKVLAKKLLDLYKTPDNIDIWIGGNAEPMVERGRVGPLLACLLGRQFQQIRDGDRFWWENPGVFTEKQRDSLQKVSFSR 668
human EPO    RNLAQLSRVLKNQDLARKFLNLYGTPDNIDIWIGAIAEPLLPGARVGPLLACLFENQFRRARDGDRFWWQKRGVFTKRQRKALSRISLSR 673
human MPO    ETVGQLGTVLRNLKLARKLMEQYGTPNNIDIWMGGVSEPLKRKGRVGPLLACIIGTQFRKLRDGDRFWWENEGVFSMQQRQALAQISLPR 701
             ..      :  :  : .:: : * : ::** ::*. :*      . **  :  ::  ** : * *****::.  :   .:.      :: .

              H21
DdPoxA       VIKRTTSNIGYVQPNVFRK*-------------------------  531
goat LPO     LICDNTHITKVPL-HAFQANNYPHDFVDCSAVDKLDLSPWASREN*  712
human EPO    IICDNTGITTVSR-DIFRANIYPRGFVNCSRIPRLNLSAWRGT*--  715
human MPO    IICDNTGITTVSKNNIFMSNSYPRDFVNCSTLPALNLASWREAS*-  745
             :*  .*        . *
```

Supplemental Figure S1. Structural alignment of DdPoxA in comparison to mammalian peroxidases. Total sequence alignment of DdPoxA with goat LPO, human EPO and human MPO. Secondary structure elements of DdPoxA are highlighted in grey (α-helices) and orange (β-strands). The native signal sequence of DdPoxA is depicted in blue. Important distal residues are shown in yellow, the conserved glutamate forming the heme to protein bond is highlighted in green. The distal isoleucin of DdPoxA is depicted in pink. The conserved proximal triad is shown in red. Fully conserved residues are marked with an asterisk, a colon indicates residues with strongly similar properties, and a period shows residues with weakly similar properties.

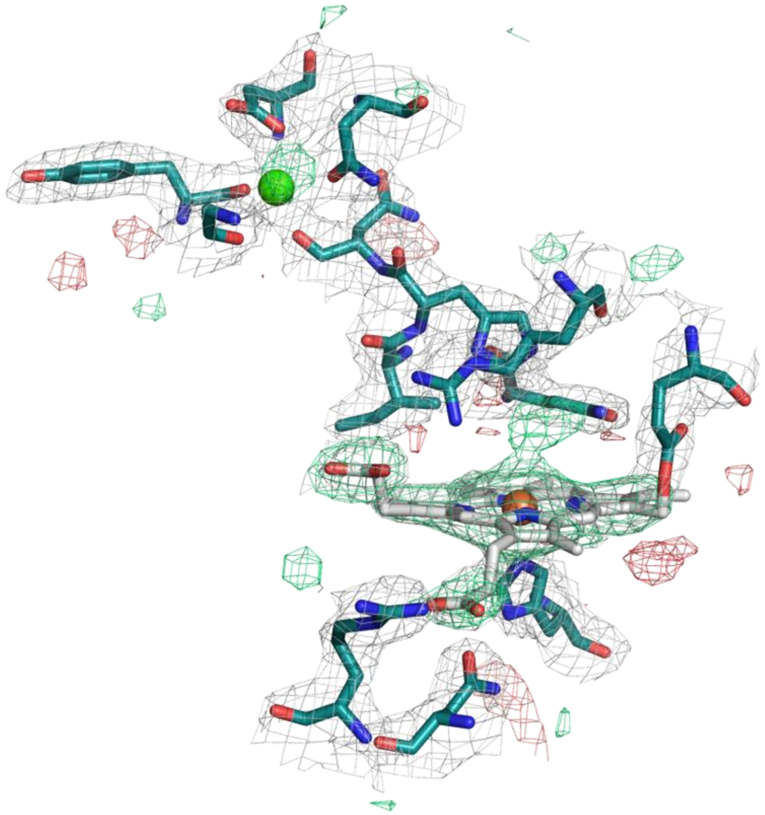

Supplemental Figure S2. OMIT map of DdPoxA active site structure. OMIT map (A) of the active site structure of DdPoxA. The 2.5 Å resolution mF_o-DF_c maps contoured at +/-3 σ positive (green) and negative (red) and $2mF_o$-DF_c maximum-likelihood omit map contoured at 1 σ (grey) are shown. Maps were calculated for a refined model with the heme atoms omitted.

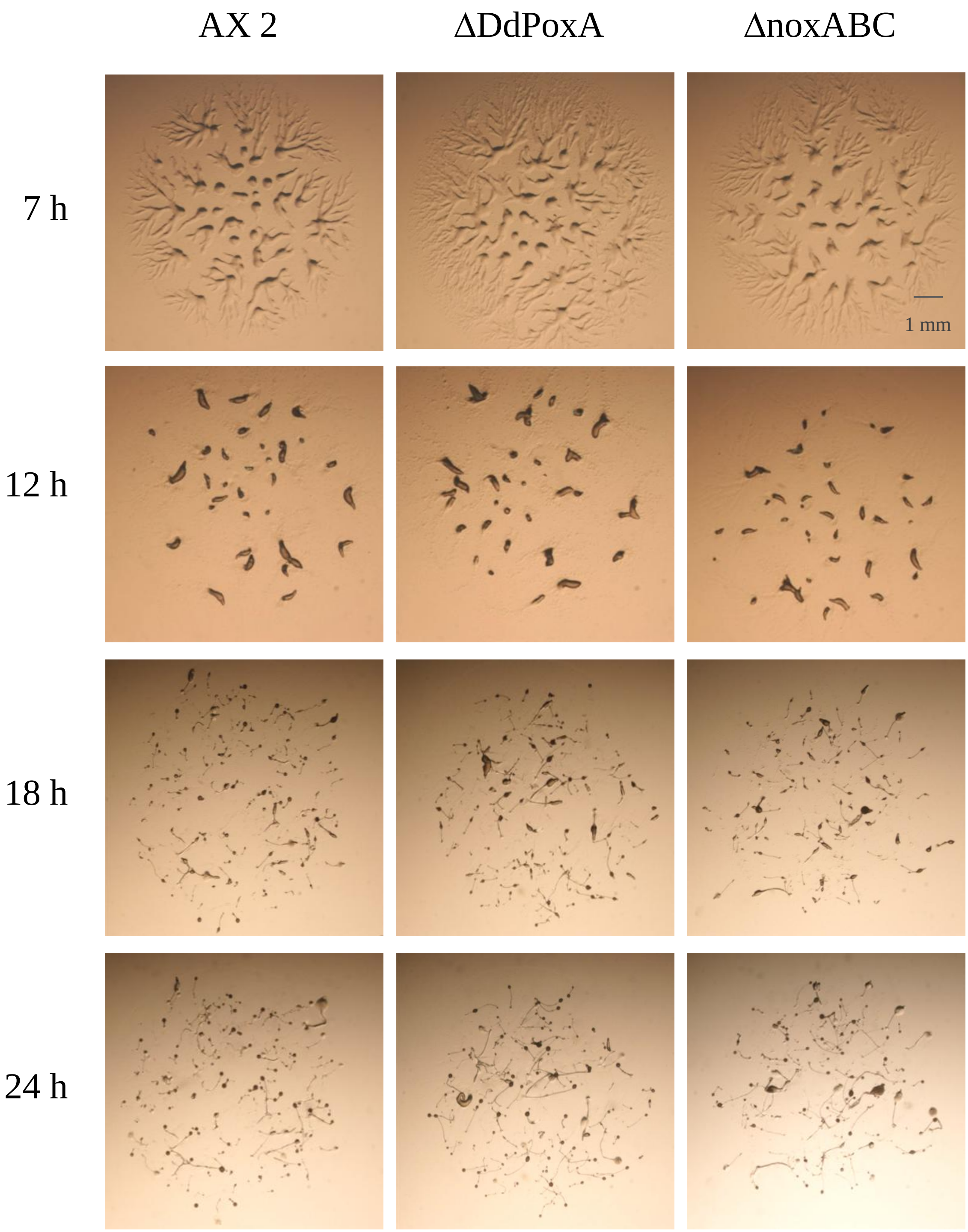

Supplemental Figure S3. Comparative cell development. Representative images of *D. discoideum* wild-type (AX2), Δ*DdpoxA* and Δ*noxABC* mutants throughout the development cycle (7, 12, 18 and 24 h after start of starvation) recorded using a stereomicroscope. Cell density in all images is 1×10^6 cells per drop.
